# Identification of novel autoantibodies in Sjögren’s disease

**DOI:** 10.3389/fimmu.2025.1524940

**Published:** 2025-02-03

**Authors:** Fiona Engelke, Petra Budde, Salvatore De Vita, Thomas Dörner, Diana Ernst, Jan Gras, Harald Heidecke, Annika Loredana Kilian, Katja Kniesch, Ann-Sophie Lindemann, Luca Quartuccio, Jacob Ritter, Kai Schulze-Forster, Benjamin Seeliger, Hans-Dieter Zucht, Torsten Witte

**Affiliations:** ^1^ Department of Rheumatology and Clinical Immunology, Hannover Medical School, Hanover, Germany; ^2^ Oncimmune Germany GmbH, Dortmund, Germany; ^3^ Department of Medicine, University of Udine, Udine, Italy; ^4^ Rheumatology and Clinical Immunology, Charité – Universitätsmedizin Berlin, Berlin, Germany; ^5^ Department of Respiratory Medicine, Hannover Medical School, Hanover, Germany; ^6^ CellTrend GmbH, Luckenwalde, Germany

**Keywords:** autoantibodies, biomarkers, connective tissue diseases, extraglandular manifestations, seronegative patients, sicca syndrome, Sjögren’s disease

## Abstract

**Introduction:**

The diagnosis of Sjögren’s disease (SjD) in patients without autoantibodies against Ro/SSA is a major challenge. We aimed to identify novel autoantibodies in SjD that may facilitate the diagnostic procedure for Ro/SSA negative SjD.

**Methods:**

IgG and IgA autoantibody reactivity of 94 potential candidate autoantigens for SjD, selected from a discovery screen of 1,629 human antigens coupled to Luminex beads and prior knowledge about potential biological relevance, were examined in serum of SjD patients (n=347) using Luminex and ELISA technology. Healthy (HC, n=118) and non-Sjögren’s sicca syndrome (NSS, n=44) individuals served as controls. To assess disease specificity, the novel autoantibodies were also measured in serum of patients with Rheumatoid Arthritis (RA, n=50), Systemic Lupus Erythematosus (SLE, n=49), and Systemic Sclerosis (SSc, n=37).

**Results:**

45 novel autoantibodies were significantly (p ≤ 0.05) more prevalent in SjD than in HC and were detected in up to 19% of the SjD cohort. The most common autoantibodies were against CCL4, M5, TMPO and OAS3. Some of the novel autoantibodies were associated with extraglandular disease manifestations, such as anti-TONSL or anti-IL6 with pulmonary involvement. We have developed a three and five marker panel for the detection of Ro/SSA negative patients, consisting of anti-FNBP4, anti-SNRPC, anti-CCL4, anti-M3 and anti-KDM6B, which had a sensitivity of up to 46% with a specificity of 95% (SjD vs. HC). Both panels discriminate these patients from HC, whereas the three-marker more effectively differentiates between Ro/SSA negative patients and NSS.

**Discussion:**

Novel autoantibodies will facilitate the diagnosis of Ro/SSA negative patients with SjD, in particular our predictive panel will be useful in the diagnosis and differentiation of these patients from healthy and NSS individuals in a clinical context. In addition, the autoantibodies may also be useful for risk stratification of extraglandular manifestations.

## 1 Introduction

Sjögren’s disease (SjD) is a chronic, heterogeneous autoimmune disorder characterized by the predominant involvement of the salivary and lachrymal glands, resulting in ocular as well as oral dryness. Other non-specific symptoms such as fatigue, myalgia and/or Raynaud’s syndrome occur in up to 70% of SjD patients, while 40% of patients with SjD have extraglandular manifestations (1). SjD may affect any organ, including lung, skin, kidney, liver, musculoskeletal and/or nervous system involvement (2, 3). Furthermore, SjD patients have an increased risk of developing B cell Non-Hodgkin lymphoma (4).

Diagnosis of SjD remains challenging due to its often non-specific symptoms and the high prevalence of reduced tear and salivary production in the general population, especially in the elderly. Infections or drugs, such as diuretics or tricyclic antidepressants, may also affect ocular and oral dryness (5). According to the current ACR/EULAR classification criteria for SjD, a histological evidence of lymphocytic foci on minor salivary gland biopsy or the presence of serological markers are required for the classification of SjD (6). Autoantibodies to Ro/SSA are the most important laboratory markers in diagnosing SjD, occurring in approximately 40-68% of patients with SjD (7). In the absence of anti-Ro/SSA antibodies, diagnosis of SjD can be much more difficult and is usually based on a salivary gland biopsy. This invasive procedure relies on the expertise of the pathologist for accurate interpretation. Recently, also salivary gland ultrasound has emerged as a potential diagnostic alternative (8). However, SjD patients without Ro/SSA antibodies are likely to be excluded from proper diagnosis and therapy, especially if a salivary gland biopsy is not performed.

We aimed to identify novel autoantibodies as biomarkers associated with SjD and hypothesized that they may be useful for the identification of patients without anti-Ro/SSA antibodies and be related with disease-specific manifestations, ultimately improving diagnosis and management for a broader range of SjD patients.

## 2 Material and methods

### 2.1 Preselection of candidate autoantigens

In a discovery phase, autoantibody reactivity was tested in a total of 134 patients with SjD meeting the ACR/EULAR classification criteria and 72 healthy controls (HC) (6). The discovery screen of IgG autoantibodies against 1,629 preselected human protein antigens using Luminex Xmap technology was performed at Oncimmune Germany GmbH (formerly Protagen AG, Dortmund, Germany). In brief, recombinant antigens were coupled to magnetic carboxylated color-coded beads (MagPlex microspheres, Luminex Corporation, Austin, Texas) as previously described in detail (9). In total, five different bead arrays were produced each comprising up to 384 antigens and including immune-relevant pathway proteins which are known to be involved in SjD and other systemic autoimmune diseases. An aliquot of each bead array was incubated with the 1:100 diluted patient serum sample. Bound antibodies were measured after incubation with a secondary phycoerythrin-labelled anti-human IgG antibody in a FlexMap3D instrument (Luminex Corporation, Austin, Texas). Data from five bead arrays were combined, the median fluorescence intensity (MFI) data were transformed to log2 values and afterwards median-centered by the sample to minimize sample and batch effects. Low reactivity antibodies were defined by calculating the 75% quantile across all samples and excluding all antigens with log2 MFI values below the threshold (log2 MFI value >10) by pre-filtering. Two group comparisons were performed using univariate tests, significance analysis of microarrays (SAM) (significance defined by p-value ≤0.05, fold-change ≥1.5, D-score ≥2) and Wilcoxon test (significance of at least one quantile defined by p-value ≤0.05, absolute span ≥1, fold-change ≥1.5) (10, 11). Candidates derived from multiple analyses were combined: SjD patients (including those with comorbidities) vs. HC, SjD patients with and without polyneuropathy vs. HC. This resulted in a total of 35 autoantibody targets for SjD, including also known SjD-related autoantibodies against Ro52/TRIM21, Ro60/TROVE2 and La/SSB.

These 35 candidates derived from the discovery screen were complemented by nine autoantigens recently identified in SjD by other groups, including autoantibodies against the muscarinic cholinergic receptors M3 and M5 as well as GRAMD1A, KLHDC8A, MAPRE1, NUP50, POLR3H, RPAP3 and TCP10L (12, 13). Further 50 antigens were selected on the basis of previous multiparametric studies in other autoimmune diseases (e. g. Rheumatoid Arthritis (RA), Systemic Lupus Erythematosus (SLE), Systemic Sclerosis (SSc)), and with potential relevance to pathogenic pathways in connective tissue diseases, as well as expression in salivary gland and glandular cells, or elevated expression in SjD-associated keratoconjunctivitis sicca (14–17). These pathways comprise proteins implicated in the immune response, complement system, Toll-like receptor signaling, lymphocyte activation such as cytokines, interferon and interferon pathway proteins according to Gene Ontology (GO) and Human Protein Atlas (HPA) annotations within the Database for Annotation, Visualization, and Integrated Discovery (DAVID). Collectively, proteins that are upregulated or expressed in tissues affected by SjD may represent potential new autoantigens. In total, 94 potential candidate autoantigens were selected for validation in SjD. A list of all candidates is provided in **Supplementary Table S1**.

### 2.2 Subjects in validation study

Serum samples of 347 patients with SjD were recruited in the Department of Rheumatology and Immunology at the Hannover Medical School (n=171), the Department of Respiratory Medicine at Hannover Medical School (n=46), the Department of Medicine/Rheumatology and Clinical Immunology at Charité in Berlin (n=72) and the Department of Medicine/Rheumatology Clinic at University of Udine (n=58). Additional serum samples from 50 patients with RA, 49 patients with SLE, 37 patients with SSc, 44 individuals with Sicca syndrome not fulfilling the 2016 ACR/EULAR classification criteria for SjD (NSS), and 118 blood donors were collected in the Department of Rheumatology and Immunology at the Hannover Medical School. SjD was not diagnosed in any of the disease controls. None of the patients or controls had previously participated in the discovery screen. The clinical and demographic characteristics of the 289 SjD patients from Hannover as well as from Berlin and from the controls (RA, SLE, SSc, NSS and HC) are shown in **Table 1**. These characteristics were not collected for the SjD samples from Udine. The high proportion of SjD patients with lung involvement in the Hannover cohort may be due to the close collaboration with the Department of Respiratory Medicine at Hannover Medical School.

**Table 1:** Characteristics of Sjögren’s disease patients (SjD, n=289) from Hannover (n=217) and Berlin (n=72), and controls (healthy controls (HC, n=118), non-Sjögren’s sicca syndrome (NSS, n=44), Rheumatoid Arthritis (RA, n=50), Systemic Lupus Erythematosus (SLE, n=49), Systemic Sclerosis (SSc, n=37)).

	SjD (n=289)		Other autoimmune diseases (n=136)			HC (n=118)	NSS (n=44)
	Hannover (n=217)	Berlin (n=72)	RA (n=50)	SLE (n=49)	SSc (n=37)		
female subjects, (%)	77.3	94.3	68	81.6	59.5	43	90
age at visit, years	61	47	58	46	57	48	54.7
age at onset, years	56	NA					
Laboratory values, (%)							
ANA ≥1:160	77.2	NA	52	100	97.2		52.3
Rheumatoid factor positive	31.8	64.2	70	6.1	5.4		4.5
Presence of Ro/SSA antibodies	61.6	80.6	2	34.7	8.1		0
Presence of La/SSB antibodies	21.4	45.8	0	6.1	2.7		0
Oral and ocular tests, (%)							
Saxon test pathological	49.3	100					54.5
Schirmer test pathological	68.5	33.3					65.9
Salivary gland biopsy, (%)							
Biopsy performed	42.3	11.4					45.5
Chisholm Mason grade ≥3	58.8	62.5					0
ESSDAI, (%)							
Constitutional symptoms	9.7	50					
Lymphadenopathy	2.7	1.4					
Glandular involvement	5.3	51.4					
Articular involvement	26.9	31.4					
Cutaneous involvement	3.4	8.6					
Pulmonary involvement	57.2	4.3					
Renal involvement	4	0					
Muscular involvement	3.4	2.9					
Peripheral nervous system involvement	11.1	5.7					
Central nervous system involvement	2.9	0					
Hematological involvement	30.2	42.9					
Biological involvement	15.7	21.4					

Provided values represent averages.

All patients were recruited over a five-year period, starting in 2016 and ending in 2021. Our study size meets the statistical power requirements for detection of meaningful differences between groups, as demonstrated by power analysis (data not shown). Serum samples were aliquoted and stored at -80°C until use. All study participants fulfilled the ACR/EULAR classification criteria for the respective disease and provided written informed consent (6, 18–20). This study adhered to the tenets of the Declaration of Helsinki and was approved by the local ethics committee (Vote Hannover Medical School ethical committee No. 5582).

### 2.3 Relative quantification of serum autoantibodies by Luminex antigen array

The 92 candidate autoantigens (excluding M3 and M5) described above were used for evaluation in patients with SjD using the Luminex bead-based antigen array. All antigens were expressed as full-length proteins in E. coli by Oncimmune Germany GmbH (Dortmund, Germany).

According to the previously described procedure, recombinant human antigens were covalently coupled to specific color-coded Luminex MagPlex microspheres (Luminex Corporation, Austin, Texas) based on a carbodiimide reaction (14). According to the manufacturer’s instructions, the optimal amount of protein for the specific coupling reaction was determined, on average 10 µg per million microspheres (21). Coupling quality was assessed by using a phycoerythrin-conjugated anti-hexahistidine tag antibody (Abcam, Cambridge, UK). Coupled autoantibodies were stored in storage buffer (PBS, 1% BSA, 0.1% Tween 20, 0.05% ProClin™ 300 (Merck KGaA, Darmstadt, Germany)) at 4°C until use.

Serum samples were diluted 1:100 in assay buffer (PBS, 0.5% BSA, 50% Low-Cross buffer (Candor Biosciences, Wangen, Germany)), to prevent cross reactivity of rheumatoid factors. The diluted samples were then added to the bead mix and incubated for 20h at 4°C. Subsequent addition of R-phycoerythrin-conjugated antibody (5 µg/ml, goat anti-human IgG/IgA (Dianova, Hamburg, Germany)) allowed detection of bound autoantibodies on a Luminex 200 instrument (Luminex Corporation, Austin, Texas). MFI values represent the IgG/IgA reactivity and were exported for data analysis.

### 2.4 Relative quantification of serum autoantibodies against M3 and M5 by ELISA

Human IgG autoantibodies against the muscarinic cholinergic receptor 3 (M3) and muscarinic cholinergic receptor 5 (M5) were detected in serum samples from SjD patients (n=347) and HC (n ≤ 55) using ELISA kits (CellTrend GmbH, Luckenwalde, Germany) according to the manufacturer’s instructions. Briefly, M3- or M5 coated polystyrene plates were incubated with samples of a 1:100 serum dilution at 4°C for 2h and additionally with horseradish peroxidase-labeled goat anti-human IgG for 1h. The autoantibody titer was estimated as arbitrary units (U) considering the standard curve of five standards ranging from 2.5 to 40 U/ml.

### 2.5 Data analysis

#### 2.5.1 Univariate analysis

The assessment of patient clinical data and the identification of candidate autoantigens were performed in a blinded manner to ensure impartial analysis. Protein interactions of the autoantibody targets were explored with STRING database (22).

All measured MFI data were log2 transformed and sample-wise median-centered to minimize variance in intensities and plate effects. Mann-Whitney U-test as a statistical approach was used to compare differences between antigen profiles in SjD and HC using R 4.2.1 (R Foundation, Vienna, Austria), p-values below 0.05 were considered as statistically significant.

Data were binarized by defining the 98% quantile of the intensity values of HC and applying this value as a cut-off for SjD samples. Higher cut-offs were applied, if the specificity in HC could be increased without changing the sensitivity in SjD. Fisher’s exact test was performed as statistical method to compare differences in antigen profiles between SjD and HC. P-values below 0.05 were considered statistically significant.

#### 2.5.2 Multivariate analysis

Autoantibody patterns were assessed by principal component analysis (PCA) using the factoextra package in R, with visualization of the first two components by mixOmics package (23, 24). To create a panel to identify seronegative SjD patients, data from multiple IgG autoantibodies were combined and SjD was predicted if any antibody was positive in the binarized data. Markers were selected using forward feature-selection, meaning the most common antigen in the seronegative subgroup were selected and successively supplemented with markers having high sensitivity, high specificity and low co-prevalence to the previous marker(s). This was done using the caret package in R (25). Binarized reactivity of individual donors for all markers in the panel were visualized by graphical heatmaps using the ComplexHeatmap package in R (26, 27).

The association between antigen and clinical data or disease manifestations was evaluated by logistic regression in dichotomous way using R’s generalized linera model (glm) function (28). Subsequently, p-values were adjusted by Benjamini-Hochberg procedure to decrease false discovery rates and to generate corrected q-values. Only coefficients greater than 0.45 were considered, ensuring that the reported association were both statistically robust and practically meaningful. Due to missing clinical data, we performed a complete-case analysis in our study, meaning all patients with missing values were excluded.

## 3 Results

### 3.1 Autoantibody reactivity profile in SjD

IgG and IgA autoantibody reactivity were evaluated to confirm the previous results from the discovery screen in a validation set of 347 SjD patients. 33 of the 35 candidate autoantigens identified in the discovery screen could be verified in our validation procedure with significantly increased levels of IgG or IgA antibodies in SjD compared to HC (Mann-Whitney test; **Supplementary Tables 1, 2**). As expected, autoantibodies against Ro52/TRIM21, Ro60/TROVE2 and La/SSB were most common in the whole SjD group. The results of the Ro/SSA classification of patients by the Luminex antigen array were comparable to those obtained in routine diagnostics, indicating the reliability of the measurement (**Supplementary Figure S1**). Of the additional potential marker candidates identified following our original discovery screen, 42 out of 59 were confirmed across the three SjD cohorts, demonstrating significantly different autoantibody reactivity compared to HC (Mann-Whitney test; **Supplementary Tables 1, 2**). Taken together, we found 72 novel antigen targets in our validation screen (**Supplementary Table S1**). Using the STRING database, these novel autoantibodies in SjD belong mainly to immune- and inflammatory-specific pathways (**Supplementary Figure S2**, **Supplementary Table S3**).

**Figure 1:**
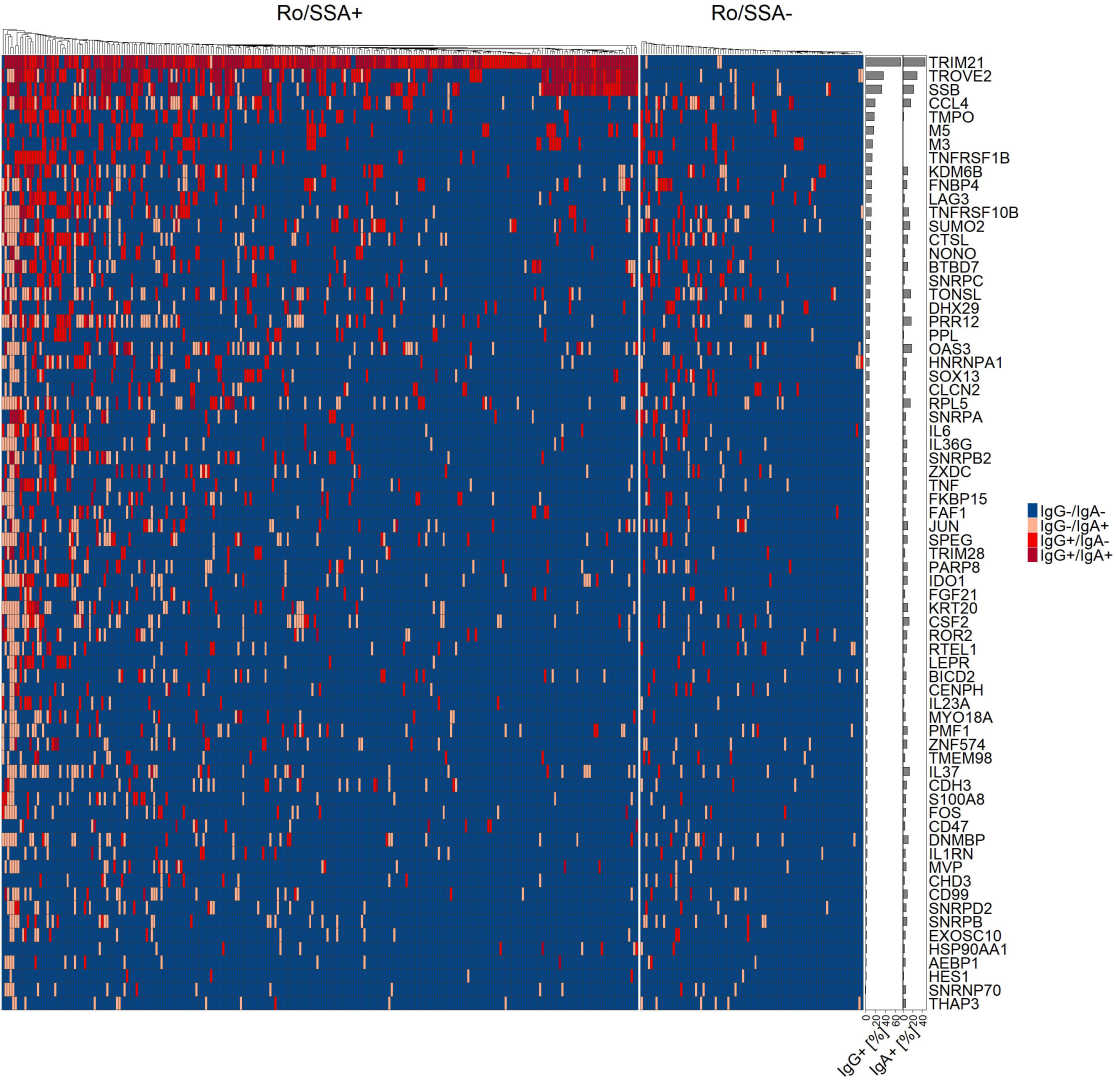
Heatmap of binarized IgG and IgA autoantibody reactivity of 72 antigens in primary Sjögren’s disease (SjD, n=347), separated into Ro/SSA positive (n=257) and negative (n=90) SjD patients. Positivity of individual patients for single or both isotypes is indicated by color code. Patients were ordered by unsupervised hierarchical cluster analysis and prevalence of each antigen in all SjD patients in percent have been added as bar chart.

Using cut-off values for autoantibody positivity that allowed a maximum of 2% positive cases in HC, 45 of the 72 novel identified antigen targets showed a significant increase (Fisher’s exact test) and were present in up to 19% of SjD patients (**Figure 1**; **Supplementary Table S2**). More specifically, the most common reactive targets in SjD were IgG autoantibodies against CCL4 (18.7%), TMPO (17.6%) and M5 (16.7%), whereas the highest IgA reactivity was found for anti-OAS3 (17.6%), anti-PRR12 (16.7%) and anti-CCL4 (15.9%). Furthermore, our data showed that several novel autoantibodies were present in multiple different autoimmune diseases or in the subgroup of NSS, while some autoantibodies appear to be increased in SjD (**Supplementary Table S2**). For example, autoantibodies against SUMO2 showed a more than two-fold higher prevalence in SjD compared to other diseases and individuals with sicca symptomatic.

In addition, PCA confirmed that the global data distribution was comparable between the three SjD cohorts from different study centers (**Supplementary Figure S3**), and all SjD patients showed an autoantibody profile almost identical to that of HC.

### 3.2 Multivariate analysis in Ro/SSA negative subjects

To address the unmet clinical need to diagnose SjD patients lacking autoantibodies to Ro/SSA, we performed a multivariate analysis to evaluate the association of autoantibodies in this subcohort. PCA revealed similar global autoantibody patterns between patients with and without autoantibodies to Ro/SSA (**Supplementary Figure S3**).

Individual novel autoantibodies were found in up to 13% of SjD patients without anti-Ro/SSA (n=90). Anti-M3 (12.2%), anti-FNBP4 (12.2%) and anti-CLCN2 (12.2%) showed high IgG reactivity in this SjD subcohort, whereas the most frequent IgA reactive targets were anti-SUMO2 (13.3%), anti-OAS3 (12.2%) and anti-CCL4 (12.2%; **Figure 1**; **Supplementary Table S2**). Seven other non-Ro/SSA associated autoantibodies described in the literature by Longobardi et al. were investigated in our Ro/SSA negative SjD cohort using the Luminex antigen array and were found in 0-6.1% of the patients, showing a lower prevalence compared to previous proteomic array results (**Supplementary Table S2**) (12).

**Figure 2:**
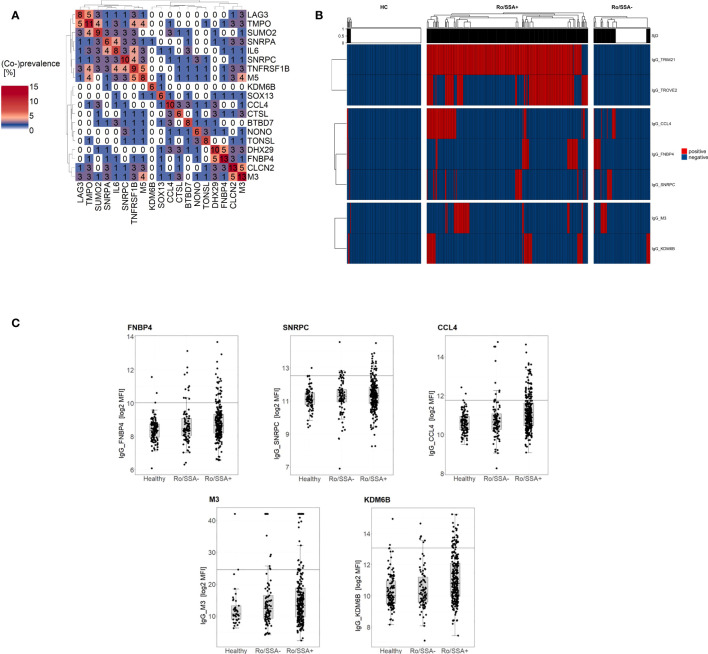
Combination of complementary autoantibodies into a panel increases diagnostic capability for identification of Ro/SSA negative SjD patients (n=90). **(A)** Co-prevalence heatmap of common IgG autoantibodies in seronegative SjD patients. The numbers in the cells represent the percentage of positive patients, for individual antibodies (diagonal) and the co-prevalence of two-marker-combinations. **(B)** Heatmap of binarized autoantibody reactivity of selected IgG antigens in Ro/SSA positive (n=257) and negative (n=90) SjD patients as well as healthy controls (n=118). The heatmap color is related to the binary outcome in antibody measurement for each patient. Patients within groups were ordered by unsupervised hierarchical cluster analysis and top annotation shows SjD panel prediction outcome, with black indicating a SjD diagnosis. **(C)** Box and whisker plots showing the log2 MFI of panel marker (FNBP4, SNRPC, CCL4, M3, KDM6B) in individual sample groups (HC, Ro/SSA-, Ro/SSA+). Horizontal lines indicate cut-off for data binarization..

Combining several complementary biomarkers can enhance the sensitivity of patient diagnosis. Therefore, we combined commonly recognized IgG antigens in seronegative SjD, by calculating the co-prevalence of these autoantibodies to assess how many SjD patients could be collectively detected by their combined presence. Since IgA antibodies are associated with disease activity and may subsequently vary over time, the panel was generated with IgG autoantibodies selected for their high sensitivity and low co-prevalence among markers using forward feature selection (**Figure 2A**). With different aims regarding diagnostic accuracy, we identified two panels combining different numbers of autoantibodies (**Figure 2B**). Anti-FNBP4, anti-SNRPC and anti-CCL4 as a combined panel identified 30% of Ro/SSA negative SjD patients. This panel had a specificity of 97% for distinguishing Ro/SSA negative patients from healthy subjects and 95% for distinguishing SjD from NSS. It may hold great promise for assessing patients with sicca symptoms in a clinical context, helping to determine whether they have SjD or whether their symptoms are related to NSS. Adding anti-M3 and anti-KDM6B to the initial panel increased the sensitivity to 46% with 95% specificity (SjD vs. HC; 84% specificity for SjD vs. NSS). Given to its higher sensitivity, this five-marker panel shows promise in detecting more Ro/SSA negative cases, especially when the initial focus is on distinguishing SjD from HC. The relative levels for each panel marker are shown in **Figure 2C**.

### 3.3 Association of clinical characteristics with autoantibody reactivity

To assess the clinical relevance of the antigen targets in SjD, both known and novel autoantibodies were investigated for potential correlation with laboratory parameters (ANA, hypergammaglobulinemia, rheumatoid factor), markers of glandular involvement (pathological saliva and tear production, histopathological salivary gland biopsy) and clinical manifestations (selected ESSDAI domains and disease activity) in 289 SjD patients from Hannover and Berlin.

**Figure 3:**
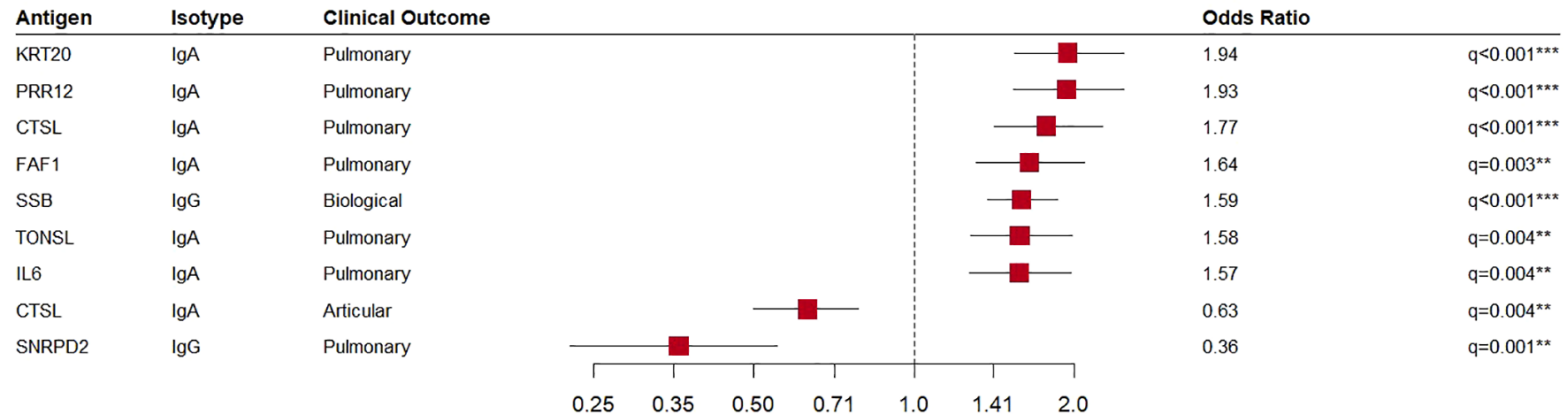
Association of autoantibodies in primary Sjögren’s disease patients (SjD, n=289) with clinical characteristics. Forest plot of logistic regression results for antibodies with significant association (p-value ≤ 0.05) and meaningful effect size (|coefficient| ≥ 0.45).

Consistent with previous studies, our data showed a positive correlation of the IgG antibodies anti-Ro60/TROVE2 and anti-La/SSB with ANA, rheumatoid factor as well as hypergammaglobulinemia (**Supplementary Table S4**) (29). A few of the autoantibodies in SjD were associated with rheumatoid factor and hypergammaglobulinemia (**Supplementary Table S4**). The association of IgA and IgG antibodies with high disease activity and selected ESSDAI domains (constitutional, glandular, articular, pulmonary, CNS, hematological, biological) was investigated in relation to clinical manifestations (**Supplementary Table S4**). Regression analyses with the ESSDAI and ESSPRI total score, showed no correlations with the novel autoantibodies in SjD. The IgA autoantibody reactivity of CTSL was negatively associated with the articular domain of the ESSDAI (**Figure 3**). Significant correlations were also observed for pulmonary involvement. Specifically, IgA autoantibodies to KRT20, PRR12, CTSL, FAF1, TONSL and IL6 were positively correlated with this organ involvement, whereas the IgG autoantibody reactivity to SNRPD2 was elevated in the absence of pulmonary manifestation. The positive correlations were supported by dot plots, in which increased activity in lung involvement is associated with higher autoantibody reactivity (**Supplementary Figure S4**).

## 4 Discussion

In this study, we identified novel autoantibodies in serum of SjD using Luminex bead-based antigen arrays or ELISA (M3 and M5), with a focus on distinguishing SjD patients from HC and in identifying the subset of patients lacking anti-Ro/SSA antibodies. Alongside the well-established autoantibodies against Ro52/TRIM21, Ro60/TROVE2 and La/SSB, several potential and novel autoantibodies identified in a pre-screen and informed by prior knowledge were verified with a frequency of up to 19% in our SjD validation cohort.

Consistent with previous studies, serum autoantibodies against M3 and M5 were also detected in a large proportion of our SjD patients (30). The previously published high prevalence of novel autoantibodies against GRAMD1A, KLHDC8A, MAPRE1, NUP50, POLR3H, RPAP3 and TCP10L using a planar array could not be confirmed in this study (12). This may be a consequence of different methods (planar microarray vs. Luminex Xmap technology). While planar microarrays are well-suited for broad autoantibody discovery due to their high antigen capacity, the flexible array design of the Luminex Xmap technology offers an alternative for designing customized panels to validate specific markers with high-throughput capacity in follow-up studies. The reliability of the Luminex Xmap technology for detecting autoantibodies is demonstrated by its comparable performance in measuring Ro/SSA autoantibodies. This was further supported by key findings showing correlations with ANA, rheumatoid factor and hypergammaglobulinemia, as published previously (29).

Targeting the unmet clinical need to diagnose SjD patients without antibodies to Ro/SSA, we have combined IgG autoantibodies into two panels that address different diagnostic purposes. The three-marker panel (anti-FNBP4, anti-SNRPC and anti-CCL4) is able to detect one third of Ro/SSA negative patients and to distinguish between SjD, HC and NSS with a high specificity of up to 97%. This combination will be very helpful in identifying Ro/SSA negative SjD patients from those with sicca syndrome but no underlying SjD. The five-marker panel (three-marker panel, anti-M3 and anti-KDM6B) showed higher sensitivity and predicted 46% of anti-Ro/SSA negative SjD patients with a specificity of 95% (SjD vs. HC). Despite the lower specificity of 84% in differentiation between SjD and NSS, the increased sensitivity makes it possible to detect a broader range of cases in comparison to the three-marker panel. Therefore, the five-marker panel may be of added value in clinical practice, especially in patients, who do not complain of sicca symptoms. Notably, 25% of our Ro/SSA negative patients identified solely by the five-marker panel did not complain of sicca symptoms (determined by ESSPRI). Consequently, this may facilitate earlier and more accurate identification of SjD in patients where conventional diagnostic criteria may fail. However, the panel size will depend on the clinical objective, such as the diagnostic accuracy (increasing sensitivity or improving specificity).

The sensitivity appears to be low for both of the panels. However, achieving 100% sensitivity is not feasible. It is important to note, that the Ro/SSA negative subset was defined based on salivary gland biopsies. The specificity of the histological results is at best only 90% and even lower in older individuals (31). Therefore, there are likely individuals with an improper diagnosis of SjD in our Ro/SSA negative subset and it is not realistic to identify novel markers for 100% of these patients.

Furthermore, some of the autoantibodies mentioned in the panel, as well as autoantibodies commonly found in SjD, are also present in other autoimmune diseases (RA, SLE and SSc). As Ro/SSA autoantibodies can occur in other autoimmune diseases, these and our proposed panel could be highly relevant in the diagnosis of SjD where non-specific symptoms pose a challenge. The main aim in clinical use is to confirm SjD and distinguish it from HC and NSS, even if the presence of autoantibodies overlaps with other diseases. As a result, patients diagnosed with SjD will benefit from early and targeted treatment. Management of potential cross-reactivity with other autoimmune diseases can be addressed at later stages of diagnosis if necessary, but is not the main focus when the priority is to diagnose SjD. However, cross-reactivity does not reduce the clinical utility and but rather offers the advantage that the presence of autoantibodies in multiple autoimmune diseases may reflect shared pathogenic pathways, providing opportunities for broader insights into autoimmunity.

Previous studies showed that IgA autoantibodies are typically present in the early phase of the disease and are associated with disease activity when comparing with IgG autoantibodies (32–37). For instance, IgA rheumatoid factor significantly correlates with an increased disease activity by showing a positive association with dryness, complement consumption, renal manifestation and focus scoring on salivary gland biopsies in SjD (34–37). In this study, we also observed significant associations between IgA autoantibodies (anti-AEBP1, anti-CCL4, anti-SNRPB) and Chisholm Mason grade 4 salivary gland biopsy. In SjD, grade 4 lip biopsy findings are characterized by extensive lymphocytic infiltration in minor salivary glands and serve as a hallmark of high disease activity. The association of IgA autoantibodies with Chisholm Mason grade 4 biopsies suggests that IgA autoantibodies correlate with local immune activation. Due to the fact that IgA autoantibodies may be associated with disease activity, only IgG autoantibodies were initially included in the panel design. However, the simultaneous detection of IgA could provide additional insight into disease activity and potentially improve diagnostic accuracy and prognosis. The extent to which the additional detection of IgA contributes to clinical utility should be further investigated in future studies.

A further important clinical application is the use of autoantibodies to predict clinical manifestations of connective tissue diseases and to measure disease activity. Interestingly, we observed relations between novel autoantibodies and clinical manifestations. A direct functional interaction between the investigated antigens or autoantibodies is so far only known for anti-M3. The G protein-coupled receptor M3 is expressed on salivary and lachrymal glands, where autoantibodies against this target imply glandular hypofunction (30). The remaining autoantibodies with significantly high prevalence in SjD were mainly components of lymphoid tissues and involved in immune system as well as inflammatory processes, as determined by the STRING database. For example, CCL4 as a cytokine is responsible for chronic inflammatory response in exocrine glands and the expression is increased in saliva of SjD patients compared to HC without sicca symptoms (38). Consistent with our findings, autoantibodies against KDM6B, SNRPC and TMPO have also been reported in SjD or other autoimmune diseases and may be involved in hematopoiesis or epithelial tissue remodeling of salivary glands, for instance (14, 39, 40).

Several of the identified novel antibody targets are expressed extracellularly. Antibodies against these antigens, such as against LAG3, may be directly involved in disease pathogenesis. LAG3 is a transmembrane protein located on T cells and is responsible for immune homeostasis by inhibiting T cell activation, and is thus crucial for maintaining immunological self-tolerance (41). In the context of autoimmune diseases such as RA, increased numbers of LAG3 positive Treg cells in lymphoid aggregation areas induce the maturation of dendritic cells and consequently the progression of inflammation in RA (42). Our study showed that autoantibodies against LAG3 were common in autoimmune diseases compared to HC, especially in SjD and RA. Autoantibodies against this target may potentially lead to an imbalance in immune homeostasis by inducing strengthened T cell response. In human cancer such as chronic lymphocytic leukemia, antibodies targeting LAG3 are being investigated as a potential therapy by negatively regulating and inhibiting T cell proliferation (43). It will be interesting to investigate in the future, whether autoantibodies against LAG3 can suppress cancer development in rheumatic diseases.

The limitations of this study include that autoantibodies were tested against proteins expressed in E. coli, which lack the post-translational modifications found in human proteins or those produced by eukaryotic expression systems. Furthermore, autoantibody levels may be affected by medication. Drugs such as immunosuppressants may alter circulating B-lymphocytes with subsequent potential variation in serum autoantibody levels. In addition, study participants were recruited at university hospitals and is likely to represent a patient group with severe disease activity and manifestations. In particular, most of the SjD patients were recruited in Hannover, with a high prevalence of pulmonary manifestation and a low prevalence of glandular and biological involvement in comparison to other SjD cohorts described in the literature. This unusual distribution of clinical features may have influenced the associations with clinical features. Therefore, cohorts from established medical practice are needed. Furthermore, the lack of clinical data from the Udine cohort reduces the completeness of the dataset and may limit the robustness of the study’s conclusions.

In future studies, we plan to investigate the consistency or variation in autoantibody level over time to assess if the observed autoantibodies are useful to predict disease and treatment outcomes as well as disease activity. Furthermore, we would like to find out, if some of the novel autoantibodies may be particularly helpful to diagnose SjD in patients with pulmonary or polyneuropathy involvement.

In conclusion, the multiparametric detection of 45 novel autoantigens in SjD and in particular a panel of up to five autoantigens (anti-FNBP4, anti-SNRPC, anti-CCL4, anti-M3 and anti-KDM6B) offers significant potential to enhance the diagnostic process, especially in Ro/SSA negative SjD patients. This approach may reduce the need for the invasive and subjective salivary gland biopsy in a large proportion of these patients.

## Data Availability

The original contributions presented in the study are included in the article/**Supplementary Material**. Further inquiries can be directed to the corresponding author.
