# Supplementary material for: Identification of novel autoantibodies in Sjögren’s disease
Source: Front Immunol. 2025 Feb 3;16:1524940. doi: 10.3389/fimmu.2025.1524940 (PMC11830686; doi:10.3389/fimmu.2025.1524940)
Supplement: Supplementary file 1 [file DataSheet1.docx]

Supplementary Material

# Supplementary Data

**Table S1**: Significantly occurring candidate autoantigens in the discovery and validation cohorts, marked with an ‘x’.

| **Gene Symbol** | **Gene Name** | **Discovery Screen** | **Validation Screen** |
| --- | --- | --- | --- |
| AEBP1 | Adipocyte enhancer-binding protein 1 |  | x |
| BICD2 | Protein bicaudal D homolog 2 | x | x |
| BMPR2 | Bone morphogenetic protein receptor type-2 |  |  |
| BTBD7 | BTB/POZ domain-containing protein 7 | x | x |
| C1QPB | Complement component 1 Q subcomponent-binding protein, mitochondrial | x |  |
| CBLC | E3 ubiquitin-protein ligase CBL-C |  |  |
| CCL4 | C-C motif chemokine 4 |  | x |
| CD320 | CD320 antigen |  |  |
| CD47 | Leukocyte surface antigen CD47 |  | x |
| CD99 | CD99 antigen |  | x |
| CDH3 | Cadherin-3 | x | x |
| CDR2 | Cerebellar degeneration-related protein 2 |  |  |
| CENPH | Centromere protein H |  | x |
| CHD3 | Chromodomain-helicase-DNA-binding protein 3 | x | x |
| CLCN2 | Chloride channel protein 2 | x | x |
| CSF2 | Granulocyte-macrophage colony-stimulating factor |  | x |
| CTSL | Procathepsin L | x | x |
| DHX29 | ATP-dependent RNA helicase DHX29 | x | x |
| DNMBP | Dynamin-binding protein | x | x |
| EXOSC10 | Exosome complex component 10 | x | x |
| FAF1 | FAS-associated factor 1 | x | x |
| FGF21 | Fibroblast growth factor 21 |  | x |
| FKBP15 | FK506-binding protein 15 | x | x |
| FNBP4 | Formin-binding protein 4 | x | x |
| FOS | Protein c-Fos |  | x |
| GAL | Galanin/GMAP prepropeptide |  |  |
| GRAMD1A | Protein Aster-A |  | x |
| HES1 | Transcription factor HES-1 |  | x |
| HNRNPA1 | Heterogeneous nuclear ribonucleoprotein A1 | x | x |
| HSP90AA1 | Heat shock protein HSP 90-alpha | x | x |
| IDO1 | Indoleamine 2,3-dioxygenase 1 |  | x |
| IL1RL1 | Interleukin 1 receptor-like 1 |  |  |
| IL1RN | Interleukin 1 receptor antagonist |  | x |
| IL23A | Interleukin 23, alpha subunit p19 |  | x |
| IL36G | Interleukin 36, gamma |  | x |
| IL36RN | Interleukin 36 receptor antagonist |  |  |
| IL37 | Interleukin 37 |  | x |
| IL6 | Interleukin 6 | x | x |
| JUN | Jun proto-oncogene |  | x |
| KDM6B | Lysine (K)-specific demethylase 6B | x | x |
| KLHDC8A | Kelch domain-containing protein 8A |  | x |
| KRT20 | Keratin 20, type I |  | x |
| KRT73 | Keratin 73, type II |  |  |
| LAG3 | Lymphocyte-activation gene 3 |  | x |
| LEPR | Leptin receptor |  | x |
| M3 | Muscarinic acetylcholine receptor M3 |  | x |
| M5 | Muscarinic acetylcholine receptor M5 |  | x |
| MAPRE1 | Microtubule-associated protein RP/EB family member 1 |  | x |
| MVP | Major vault protein | x | x |
| MYO18A | Myosin XVIIIA |  | x |
| NONO | Non-POU domain containing, octamer-binding | x | x |
| NUMA1 | Nuclear mitotic apparatus protein 1 | x |  |
| NUP50 | Nuclear pore complex protein Nup50 |  |  |
| OAS3 | 2'-5'-oligoadenylate synthase 3 | x | x |
| PARP8 | Protein mono-ADP-ribosyltransferase PARP8 | x | x |
| PMF1 | Polyamine-modulated factor 1 |  | x |
| POLR3B | Polymerase (RNA) III (DNA directed) polypeptide B |  |  |
| POLR3H | DNA-directed RNA polymerase III subunit RPC8 |  |  |
| PPL | Periplakin | x | x |
| PRR12 | Proline rich 12 |  | x |
| PSMD10 | Proteasome (prosome, macropain) 26S subunit, non-ATPase, 10 |  |  |
| ROR2 | Receptor tyrosine kinase-like orphan receptor 2 |  | x |
| RPAP3 | RNA polymerase II-associated protein 3 |  | x |
| RPL5 | Large ribosomal subunit protein uL18 |  | x |
| RPLP2 | Ribosomal protein, large, P2 |  |  |
| RPP25 | Ribonuclease P/MRP 25kDa subunit |  |  |
| RTEL1 | Regulator of telomere elongation helicase 1 |  | x |
| S100A8 | S100 calcium binding protein A8 |  | x |
| SNRNP70 | Small nuclear ribonucleoprotein 70kDa (U1) | x | x |
| SNRPA | Small nuclear ribonucleoprotein polypeptide A | x | x |
| SNRPB | Small nuclear ribonucleoprotein polypeptides B and B1 | x | x |
| SNRPB2 | Small nuclear ribonucleoprotein polypeptide B | x | x |
| SNRPC | U1 small nuclear ribonucleoprotein C |  | x |
| SNRPD2 | Small nuclear ribonucleoprotein D2 polypeptide 16.5kDa |  | x |
| SNRPF | Small nuclear ribonucleoprotein polypeptide F |  |  |
| SOX13 | Transcription factor SOX-13 | x | x |
| SPEG | Striated muscle preferentially expressed protein kinase | x | x |
| SPP1 | Secreted phosphoprotein 1 |  |  |
| SSB | Sjogren syndrome antigen B (autoantigen La) | x | x |
| SUMO2 | Small ubiquitin-like modifier 2 |  | x |
| TCP10L | T-complex protein 10A homolog 1 |  | x |
| THAP3 | THAP domain-containing protein 3 | x | x |
| TMEM98 | Transmembrane protein 98 |  | x |
| TMPO | Thymopoietin | x | x |
| TNF | Tumor necrosis factor |  | x |
| TNFRFS1B | Tumor necrosis factor receptor superfamily, member 1B |  | x |
| TNFRSF10B | Tumor necrosis factor receptor superfamily, member 10b |  | x |
| TONSL | Tonsoku-like, DNA repair protein |  | x |
| TRIM21 | Tripartite motif containing 21 | x | x |
| TRIM28 | Tripartite motif containing 28 |  | x |
| TRNAU1AP | TRNA selenocysteine 1 associated protein 1 |  |  |
| TROVE2 | TROVE domain family, member 2 | x | x |
| ZNF574 | Zinc finger protein 574 | x | x |
| ZXDC | ZXD family zinc finger C |  | x |

**Table S2**: Prevalence of IgG and IgA autoantibody reactivity in primary Sjögren’s disease (SjD, n=347), separated into all and Ro/SSA negative SjD patients (n=90), and in healthy controls (HC, n=118), non-Sjögren’s sicca syndrome (NSS, n=44) as well as other autoimmune diseases, including Rheumatoid Arthritis (RA, n=50), Systemic Lupus Erythematosus (SLE, n=49), Systemic Sclerosis (SSc, n=37). P-value refers to a two group comparison between all SjD patients and HC using Mann-Whitney U-test.

|  |  |  |  | **percent positive patients^c^** | | | | | |
| --- | --- | --- | --- | --- | --- | --- | --- | --- | --- |
|  |  |  |  | **SjD** | |  |  |  |  |
| **Antigen** | **p-value^a^** | **p-value^b^** | **HC** | **all** | **Ro/SSA negative** | **NSS** | **SLE** | **RA** | **SSc** |
| IgG_AEBP1 | 2.56E-01 | 6.46E-01 | 1.7 | 1.2 | 1.1 | 2.3 | 2.0 | 8.0 | 0.0 |
| IgA_AEBP1 | 5.30E-03 | 3.75E-01 | 1.7 | 3.7 | 2.2 | 4.5 | 8.2 | 18.0 | 16.2 |
| IgG_BICD2 | 1.84E-03 | 3.75E-01 | 1.7 | 3.7 | 2.2 | 2.3 | 2.0 | 4.0 | 2.7 |
| IgA_BICD2 | 2.36E-04 | 8.23E-02 | 1.7 | 6.1 | 4.4 | 9.1 | 0.0 | 8 | 2.7 |
| IgG_BTBD7 | 5.25E-02 | 5.00E-04 | 0.8 | 9.8 | 7.8 | 2.3 | 6.1 | 10 | 10.8 |
| IgA_BTBD7 | 3.03E-03 | 3.90E-03 | 1.7 | 9.5 | 6.7 | 2.3 | 6.1 | 12.0 | 5.4 |
| IgG_CCL4 | 1.30E-02 | 1.00E-04 | 1.7 | 18.7 | 10.0 | 2.3 | 18.4 | 30.0 | 5.4 |
| IgA_CCL4 | 3.66E-04 | 1.00E-04 | 1.7 | 15.9 | 12.2 | 4.5 | 6.1 | 2.0 | 2.7 |
| IgG_CD47 | 4.77E-02 | 7.39E-01 | 1.7 | 2.9 | 0.0 | 6.8 | 8.2 | 14.0 | 8.1 |
| IgA_CD47 | 7.26E-04 | 5.31E-01 | 1.7 | 3.2 | 2.2 | 2.3 | 2.0 | 4.0 | 2.7 |
| IgG_CD99 | 1.20E-01 | 1.00E+00 | 1.7 | 2.0 | 2.2 | 2.3 | 0.0 | 8.0 | 2.7 |
| IgA_CD99 | 2.49E-02 | 1.00E-02 | 1.7 | 8.6 | 6.7 | 4.5 | 4.1 | 2.0 | 5.4 |
| IgG_CDH3 | 2.28E-02 | 7.39E-01 | 1.7 | 2.9 | 1.1 | 0.0 | 4.1 | 6.0 | 2.7 |
| IgA_CDH3 | 1.12E-01 | 5.39E-02 | 1.7 | 2.6 | 3.3 | 6.8 | 8.2 | 14 | 5.4 |
| IgG_CENPH | 3.40E-02 | 3.75E-01 | 1.7 | 3.7 | 5.6 | 0.0 | 0.0 | 4.0 | 2.7 |
| IgA_CENPH | 6.47E-03 | 2.07E-01 | 0.8 | 3.7 | 3.3 | 4.5 | 2.0 | 2.0 | 0.0 |
| IgG_CHD3 | 7.92E-01 | 4.60E-01 | 0.8 | 2.3 | 1.1 | 2.3 | 2.0 | 2.0 | 5.4 |
| IgA_CHD3 | 2.78E-02 | 7.38E-01 | 1.7 | 6.3 | 3.3 | 0.0 | 2.0 | 2.0 | 0.0 |
| IgG_CLCN2 | 7.44E-03 | 2.33E-02 | 1.7 | 7.5 | 12.2 | 9.1 | 6.1 | 14.0 | 10.8 |
| IgA_CLCN2 | 1.58E-04 | 3.75E-01 | 1.7 | 3.7 | 3.3 | 2.3 | 2.0 | 4.0 | 0.0 |
| IgG_CSF2 | 3.10E-02 | 3.79E-01 | 1.7 | 4.0 | 4.4 | 2.3 | 2.0 | 20.0 | 5.4 |
| IgA_CSF2 | 4.22E-03 | 2.00E-04 | 1.7 | 12.4 | 5.6 | 4.5 | 8.2 | 8.0 | 10.8 |
| IgG_CTSL | 4.66E-02 | 1.60E-03 | 1.7 | 10.7 | 8.9 | 2.3 | 0.0 | 38.0 | 0.0 |
| IgA_CTSL | 2.73E-02 | 3.90E-03 | 1.7 | 9.5 | 4.4 | 9.1 | 4.1 | 10.0 | 8.1 |
| IgG_DHX29 | 1.05E-03 | 6.20E-03 | 1.7 | 8.9 | 8.9 | 6.8 | 6.1 | 14.0 | 5.4 |
| IgA_DHX29 | 2.44E-04 | 5.34E-01 | 1.7 | 3.5 | 3.3 | 2.3 | 0.0 | 2.0 | 2.7 |
| IgG_DNMBP | 2.77E-03 | 7.38E-01 | 1.7 | 2.6 | 2.2 | 2.3 | 2.0 | 2.0 | 5.4 |
| IgA_DNMBP | 8.95E-02 | 2.50E-03 | 1.7 | 10.1 | 10.0 | 9.1 | 8.2 | 4.0 | 2.7 |
| IgG_EXOSC10 | 4.28E-01 | 3.45E-01 | 0.0 | 1.7 | 3.3 | 4.5 | 6.1 | 6.0 | 2.7 |
| IgA_EXOSC10 | 1.07E-03 | 3.75E-01 | 1.7 | 3.7 | 0.0 | 2.3 | 2.0 | 2.0 | 0.0 |
| IgG_FAF1 | 2.72E-03 | 8.23E-02 | 1.7 | 6.1 | 3.3 | 0.0 | 6.1 | 0.0 | 0.0 |
| IgA_FAF1 | 2.94E-02 | 3.75E-01 | 1.7 | 3.7 | 2.2 | 0.0 | 4.1 | 6.0 | 2.7 |
| IgG_FGF21 | 4.74E-02 | 8.47E-02 | 0.8 | 4.6 | 3.3 | 4.5 | 4.1 | 18.0 | 0.0 |
| IgA_FGF21 | 6.09E-02 | 5.34E-01 | 1.7 | 3.5 | 2.2 | 6.8 | 4.1 | 0.0 | 0.0 |
| IgG_FKBP15 | 3.96E-02 | 8.23E-02 | 1.7 | 6.1 | 4.4 | 4.5 | 10.2 | 8.0 | 10.8 |
| IgA_FKBP15 | 6.88E-03 | 8.13E-02 | 1.7 | 5.8 | 3.3 | 4.5 | 6.1 | 10.0 | 10.8 |
| IgG_FNBP4 | 6.63E-02 | 4.00E-04 | 1.7 | 12.1 | 12.2 | 2.3 | NA | NA | NA |
| IgA_FNBP4 | 7.12E-03 | 1.52E-02 | 1.7 | 8.1 | 4.4 | 2.3 | NA | NA | NA |
| IgG_FOS | 3.68E-03 | 7.39E-01 | 1.7 | 2.9 | 2.2 | 0.0 | 4.1 | 0.0 | 8.1 |
| IgA_FOS | 2.27E-02 | 1.79E-01 | 1.7 | 4.9 | 2.2 | 6.8 | 4.1 | 4.0 | 0.0 |
| IgG_GRAMD1A | 2.29E-02 | 2.20E-03 | 0.8 | 8.5 | 6.1 | 4.5 | NA | NA | NA |
| IgA_GRAMD1A | 1.00E-04 | 1.00E+00 | 0.8 | 0.8 | 1.0 | 0.0 | NA | NA | NA |
| IgG_HES1 | 5.72E-01 | 1.00E+00 | 0.8 | 0.9 | 0.0 | 0.0 | 2.0 | 2.0 | 2.7 |
| IgA_HES1 | 3.05E-03 | 5.75E-01 | 0.0 | 0.9 | 1.1 | 0.0 | 0.0 | 0.0 | 0.0 |
| IgG_HNRNPA1 | 2.89E-02 | 2.33E-02 | 1.7 | 7.5 | 4.4 | 2.3 | 18.4 | 24.0 | 5.4 |
| IgA_HNRNPA1 | 9.65E-03 | 1.37E-02 | 0.8 | 6.6 | 5.6 | 13.6 | 4.1 | 4.0 | 2.7 |
| IgG_HSP90AA1 | 7.55E-03 | 1.00E+00 | 1.7 | 1.7 | 1.1 | 2.3 | 0.0 | 2.0 | 2.7 |
| IgA_HSP90AA1 | 2.19E-03 | 1.00E+00 | 1.7 | 2.3 | 1.1 | 6.8 | 2.0 | 4.0 | 2.7 |
| IgG_IDO1 | 7.19E-01 | 2.66E-01 | 1.7 | 4.6 | 1.1 | 0.0 | 0.0 | 10.0 | 0.0 |
| IgA_IDO1 | 2.04E-02 | 9.70E-03 | 1.7 | 8.4 | 0.0 | 6.8 | 4.1 | 0.0 | 0.0 |
| IgG_IL1RN | 9.12E-01 | 7.38E-01 | 1.7 | 2.6 | 2.2 | 2.3 | 4.1 | 4.0 | 2.7 |
| IgA_IL1RN | 7.95E-01 | 1.30E-01 | 0.8 | 4.0 | 4.4 | 6.8 | 2.0 | 4.0 | 0.0 |
| IgG_IL23A | 5.15E-01 | 3.75E-01 | 1.7 | 3.7 | 0.0 | 2.3 | 2.0 | 14.0 | 0.0 |
| IgA_IL23A | 3.39E-02 | 1.00E+00 | 1.7 | 1.7 | 2.2 | 6.8 | 0.0 | 0.0 | 0.0 |
| IgG_IL36G | 2.79E-02 | 3.55E-02 | 1.7 | 6.9 | 2.2 | 6.8 | 12.2 | 32.0 | 0.0 |
| IgA_IL36G | 1.52E-04 | 2.35E-02 | 1.7 | 7.2 | 5.6 | 4.5 | 4.1 | 2.0 | 2.7 |
| IgG_IL37 | 2.26E-01 | 3.04E-01 | 0.8 | 2.9 | 1.1 | 0.0 | 4.1 | 12.0 | 5.4 |
| IgA_IL37 | 3.11E-03 | 1.00E-04 | 1.7 | 13.3 | 5.6 | 9.1 | 8.2 | 2.0 | 8.1 |
| IgG_IL6 | 1.59E-01 | 7.00E-04 | 0.0 | 7.2 | 6.7 | 4.5 | 4.1 | 8.0 | 2.7 |
| IgA_IL6 | 2.72E-03 | 3.75E-01 | 1.7 | 3.7 | 2.2 | 0.0 | 4.1 | 2.0 | 2.7 |
| IgG_JUN | 2.98E-04 | 1.22E-01 | 1.7 | 5.2 | 3.3 | 6.8 | 14.3 | 16.0 | 2.7 |
| IgA_JUN | 1.42E-02 | 3.90E-03 | 1.7 | 9.5 | 8.9 | 6.8 | 2.0 | 2.0 | 5.4 |
| IgG_KDM6B | 4.28E-03 | 2.00E-04 | 1.7 | 12.4 | 6.7 | 9.1 | 10.2 | 0.0 | 18.9 |
| IgA_KDM6B | 4.07E-03 | 3.90E-03 | 1.7 | 9.5 | 6.7 | 4.5 | 8.2 | 0.0 | 13.5 |
| IgG_KLHDC8A | 2.00E-04 | 1.29E-01 | 0.8 | 2.8 | 1.0 | 0.0 | NA | NA | NA |
| IgA_KLHDC8A | 1.00E-04 | 5.76E-01 | 0.8 | 1.1 | 0.0 | 0.0 | NA | NA | NA |
| IgG_KRT20 | 4.91E-02 | 2.61E-01 | 1.7 | 4.3 | 0.0 | 0.0 | 8.2 | 18.0 | 5.4 |
| IgA_KRT20 | 2.55E-03 | 3.90E-03 | 1.7 | 9.5 | 3.3 | 0.0 | 6.1 | 10.0 | 5.4 |
| IgG_LAG3 | 1.88E-01 | 4.00E-04 | 1.7 | 11.8 | 7.8 | 13.6 | 6.1 | 24.0 | 0.0 |
| IgA_LAG3 | 1.29E-03 | 1.00E+00 | 1.7 | 2.0 | 0.0 | 0.0 | 4.1 | 2.0 | 0.0 |
| IgG_LEPR | 3.93E-02 | 2.57E-02 | 0.0 | 4.0 | 1.1 | 2.3 | 6.1 | 2.0 | 5.4 |
| IgA_LEPR | 3.24E-01 | 6.86E-01 | 0.8 | 2.0 | 0.0 | 0.0 | 0.0 | 0.0 | 0.0 |
| IgG_MVP | 4.35E-01 | 1.00E+00 | 1.7 | 2.3 | 0.0 | 2.3 | 0.0 | 4.0 | 2.7 |
| IgA_MVP | 2.04E-02 | 1.22E-01 | 1.7 | 5.5 | 3.3 | 2.3 | 16.3 | 10.0 | 8.1 |
| IgG_MYO18A | 1.01E-03 | 5.34E-01 | 1.7 | 3.5 | 1.1 | 4.5 | 2.0 | 6.0 | 0.0 |
| IgA_MYO18A | 1.49E-02 | 2.61E-01 | 1.7 | 4.3 | 0.0 | 9.1 | 2.0 | 4.0 | 2.7 |
| IgG_NONO | 4.80E-05 | 1.60E-03 | 1.7 | 10.4 | 5.6 | 6.8 | 12.2 | 10.0 | 0.0 |
| IgA_NONO | 6.95E-03 | 5.34E-01 | 1.7 | 3.5 | 1.1 | 2.3 | 0.0 | 2.0 | 2.7 |
| IgG_OAS3 | 2.73E-02 | 5.20E-03 | 0.8 | 7.5 | 3.3 | 6.8 | 4.1 | 0.0 | 5.4 |
| IgA_OAS3 | 5.65E-05 | 1.00E-04 | 1.7 | 17.6 | 12.2 | 18.2 | 10.2 | 8.0 | 21.6 |
| IgG_PARP8 | 1.74E-01 | 1.79E-01 | 1.7 | 4.9 | 4.4 | 4.5 | 8.2 | 6.0 | 2.7 |
| IgA_PARP8 | 1.82E-02 | 9.70E-03 | 1.7 | 8.4 | 6.7 | 11.4 | 10.2 | 10.0 | 2.7 |
| IgG_PMF1 | 1.53E-02 | 3.11E-01 | 0.8 | 3.2 | 1.1 | 0.0 | 6.1 | 6.0 | 5.4 |
| IgA_PMF1 | 3.94E-06 | 6.20E-03 | 1.7 | 8.9 | 4.4 | 4.5 | 2.0 | 10.0 | 16.2 |
| IgG_PPL | 1.16E-01 | 1.51E-02 | 1.7 | 7.8 | 3.3 | 6.8 | 16.3 | 16.0 | 2.7 |
| IgA_PPL | 1.65E-03 | 6.05E-01 | 1.7 | 0.9 | 1.1 | 0.0 | 0.0 | 0.0 | 0.0 |
| IgG_PRR12 | 1.53E-01 | 1.51E-02 | 1.7 | 7.8 | 3.3 | 6.8 | 6.1 | 6.0 | 0.0 |
| IgA_PRR12 | 1.04E-02 | 1.00E-04 | 1.7 | 16.7 | 10.0 | 4.5 | 10.2 | 6.0 | 13.5 |
| IgG_ROR2 | 5.04E-01 | 3.79E-01 | 1.7 | 4.0 | 1.1 | 0.0 | 0.0 | 6.0 | 0.0 |
| IgA_ROR2 | 1.03E-02 | 1.51E-02 | 1.7 | 7.8 | 5.6 | 4.5 | 0.0 | 8.0 | 10.8 |
| IgG_RPL5 | 1.76E-01 | 2.35E-02 | 1.7 | 7.2 | 2.2 | 0.0 | 2.0 | 0.0 | 2.7 |
| IgA_RPL5 | 3.12E-09 | 1.00E-04 | 1.7 | 15.0 | 8.9 | 13.6 | 10.2 | 8.0 | 16.2 |
| IgG_RTEL1 | 2.85E-01 | 1.30E-01 | 0.8 | 4.0 | 5.6 | 2.3 | 4.1 | 10.0 | 10.8 |
| IgA_RTEL1 | 5.36E-02 | 5.39E-02 | 1.7 | 6.3 | 6.7 | 4.5 | 8.2 | 4.0 | 2.7 |
| IgG_S100A8 | 2.56E-01 | 7.39E-01 | 1.7 | 2.9 | 1.1 | 0.0 | 0.0 | 6.0 | 0.0 |
| IgA_S100A8 | 1.08E-02 | 1.22E-01 | 1.7 | 5.2 | 1.1 | 2.3 | 2.0 | 4.0 | 5.4 |
| IgG_SNRNP70 | 5.40E-01 | 2.68E-01 | 1.7 | 0.6 | 1.1 | 0.0 | 4.1 | 14.0 | 0.0 |
| IgA_SNRNP70 | 1.30E-03 | 2.66E-01 | 1.7 | 4.6 | 3.3 | 0.0 | 2.0 | 0.0 | 0.0 |
| IgG_SNRPA | 8.21E-03 | 2.35E-02 | 1.7 | 7.2 | 6.7 | 0.0 | 26.5 | 8.0 | 0.0 |
| IgA_SNRPA | 2.41E-03 | 1.79E-01 | 1.7 | 4.9 | 3.3 | 4.5 | 2.0 | 2.0 | 0.0 |
| IgG_SNRPB | 2.13E-03 | 1.00E+00 | 1.7 | 1.7 | 1.1 | 2.3 | 26.5 | 30.0 | 0.0 |
| IgA_SNRPB | 2.82E-03 | 2.33E-02 | 1.7 | 7.5 | 8.9 | 4.5 | 4.1 | 2.0 | 2.7 |
| IgG_SNRPB2 | 2.49E-03 | 3.55E-02 | 1.7 | 6.9 | 4.4 | 4.5 | 12.2 | 6.0 | 0.0 |
| IgA_SNRPB2 | 3.93E-02 | 5.54E-02 | 1.7 | 6.6 | 2.2 | 4.5 | 6.1 | 12.0 | 10.8 |
| IgG_SNRPC | 2.34E-01 | 5.00E-04 | 0.8 | 9.8 | 11.1 | 0 | 18.4 | 6 | 8.1 |
| IgA_SNRPC | 8.60E-03 | 1.99E-01 | 0.0 | 2.0 | 1.1 | NA | 8.2 | 6 | 16.2 |
| IgG_SNRPD2 | 4.88E-02 | 1.00E+00 | 1.7 | 2.0 | 2.2 | 0.0 | 12.2 | 18.0 | 0.0 |
| IgA_SNRPD2 | 4.73E-01 | 8.13E-02 | 1.7 | 5.8 | 2.2 | 4.5 | 4.1 | 6.0 | 0.0 |
| IgG_SOX13 | 1.69E-02 | 2.33E-02 | 1.7 | 7.5 | 6.7 | 13.6 | 4.1 | 4.0 | 8.1 |
| IgA_SOX13 | 4.22E-04 | 2.66E-01 | 1.7 | 4.6 | 1.1 | 0.0 | 0.0 | 8.0 | 0.0 |
| IgG_SPEG | 2.97E-01 | 1.22E-01 | 1.7 | 5.2 | 3.3 | 2.3 | 2.0 | 2.0 | 0.0 |
| IgA_SPEG | 5.85E-03 | 9.70E-03 | 1.7 | 8.4 | 1.1 | 6.8 | 8.2 | 14.0 | 13.5 |
| IgG_SSB | 1.15E-07 | 1.00E-04 | 1.7 | 32.3 | 3.3 | 4.5 | 12.2 | 4.0 | 0.0 |
| IgA_SSB | 1.35E-06 | 1.00E-04 | 1.7 | 21.6 | 7.8 | 11.4 | 6.1 | 6.0 | 8.1 |
| IgG_SUMO2 | 1.21E-02 | 1.60E-03 | 1.7 | 10.7 | 7.8 | 2.3 | 4.1 | 0.0 | 2.7 |
| IgA_SUMO2 | 3.10E-03 | 1.00E-04 | 1.7 | 13.5 | 13.3 | 13.6 | 2.0 | 4.0 | 5.4 |
| IgG_THAP3 | 6.57E-01 | 1.60E-01 | 1.7 | 0.3 | 0.0 | 0.0 | 0.0 | 8.0 | 0.0 |
| IgA_THAP3 | 4.61E-02 | 2.66E-01 | 1.7 | 4.6 | 6.7 | 4.5 | 0.0 | 4.0 | 5.4 |
| IgG_TMEM98 | 1.80E-02 | 5.31E-01 | 1.7 | 3.2 | 4.4 | 2.3 | 2.0 | 4.0 | 2.7 |
| IgA_TMEM98 | 4.50E-04 | 7.39E-01 | 1.7 | 2.9 | 2.2 | 2.3 | 0.0 | 8.0 | 0.0 |
| IgG_TMPO | 2.43E-04 | 1.00E-04 | 1.7 | 17.6 | 10.0 | 18.2 | 8.2 | 4.0 | 5.4 |
| IgA_TMPO | 6.46E-04 | 1.00E+00 | 0.8 | 0.6 | 1.1 | 2.3 | 0.0 | 0.0 | 0.0 |
| IgG_TNF | 9.98E-01 | 5.39E-02 | 1.7 | 6.3 | 1.1 | 4.5 | 0.0 | 8.0 | 2.7 |
| IgA_TNF | 1.47E-02 | 5.39E-02 | 1.7 | 6.3 | 4.4 | 2.3 | 2.0 | 2.0 | 2.7 |
| IgG_TNFRSF10B | 9.36E-01 | 6.00E-04 | 1.7 | 11.2 | 5.6 | 6.8 | 8.2 | 16.0 | 0.0 |
| IgA_TNFRSF10B | 2.45E-02 | 1.00E-03 | 1.7 | 11.0 | 8.9 | 34.1 | 0.0 | 0.0 | 0.0 |
| IgG_TNFRSF1B | 1.07E-02 | 1.00E-04 | 1.7 | 13.0 | 8.9 | 11.4 | 10.2 | 24.0 | 10.8 |
| IgA_TNFRSF1B | 1.04E-02 | NA | 0.0 | 0.0 | 0.0 | 0.0 | 6.1 | 4.0 | 0.0 |
| IgG_TONSL | 3.08E-02 | 4.00E-03 | 1.7 | 9.2 | 8.9 | 20.5 | 20.4 | 0.0 | 8.1 |
| IgA_TONSL | 2.18E-02 | 1.00E-04 | 1.7 | 15.6 | 11.1 | 4.5 | 8.2 | 2.0 | 2.7 |
| IgG_TRIM21 | 2.20E-16 | 1.00E-04 | 0.0 | 70.9 | 0.0 | 0.0 | 36.7 | 14.0 | 18.9 |
| IgA_TRIM21 | 2.20E-16 | 1.00E-04 | 1.7 | 46.1 | 3.3 | 4.5 | 12.2 | 2.0 | 10.8 |
| IgG_TRIM28 | 3.13E-02 | 1.22E-01 | 1.7 | 5.2 | 1.1 | 0.0 | 4.1 | 6.0 | 5.4 |
| IgA_TRIM28 | 4.56E-01 | 5.34E-01 | 1.7 | 3.5 | 2.2 | 2.3 | 2.0 | 2.0 | 2.7 |
| IgG_TROVE2 | 2.20E-16 | 1.00E-04 | 0 | 35.7 | 0.0 | 6.8 | 20.4 | 24.0 | 0.0 |
| IgA_TROVE2 | 9.20E-09 | 1.00E-04 | 1.7 | 28.8 | 6.7 | 0.0 | 6.1 | 0.0 | 0.0 |
| IgG_ZNF574 | 8.60E-04 | 5.31E-01 | 1.7 | 3.2 | 0.0 | 0.0 | 6.1 | 2.0 | 0.0 |
| IgA_ZNF574 | 1.60E-03 | 2.33E-02 | 1.7 | 7.5 | 6.7 | 2.3 | 4.1 | 6.0 | 5.4 |
| IgG_ZXDC | 1.03E-02 | 5.39E-02 | 1.7 | 6.3 | 4.4 | 4.5 | 6.1 | 2.0 | 0.0 |
| IgA_ZXDC | 3.11E-02 | 8.40E-03 | 0.8 | 6.9 | 4.4 | 6.8 | 8.2 | 14.0 | 10.8 |
| IgG_M3 | 1.33E-02 | 1.00E-04 | 0.8 | 13.8 | 12.2 | 9.1 | NA | NA | NA |
| IgG_M5 | 4.80E-03 | 1.00E-03 | 0.8 | 16.7 | 7.8 | 0.0 | 25.0 | 0.0 | NA |

^a^p-values were calculated applying Mann-Whitney test for SjD (n=347) versus HC (n=118).

^b^p-values were calculated applying Fisher’s exact test for SjD (n=347) versus HC (n=118).

^c^Percentage of positive patients defined by the ≥98% quantile of HC as cut-off value.

**
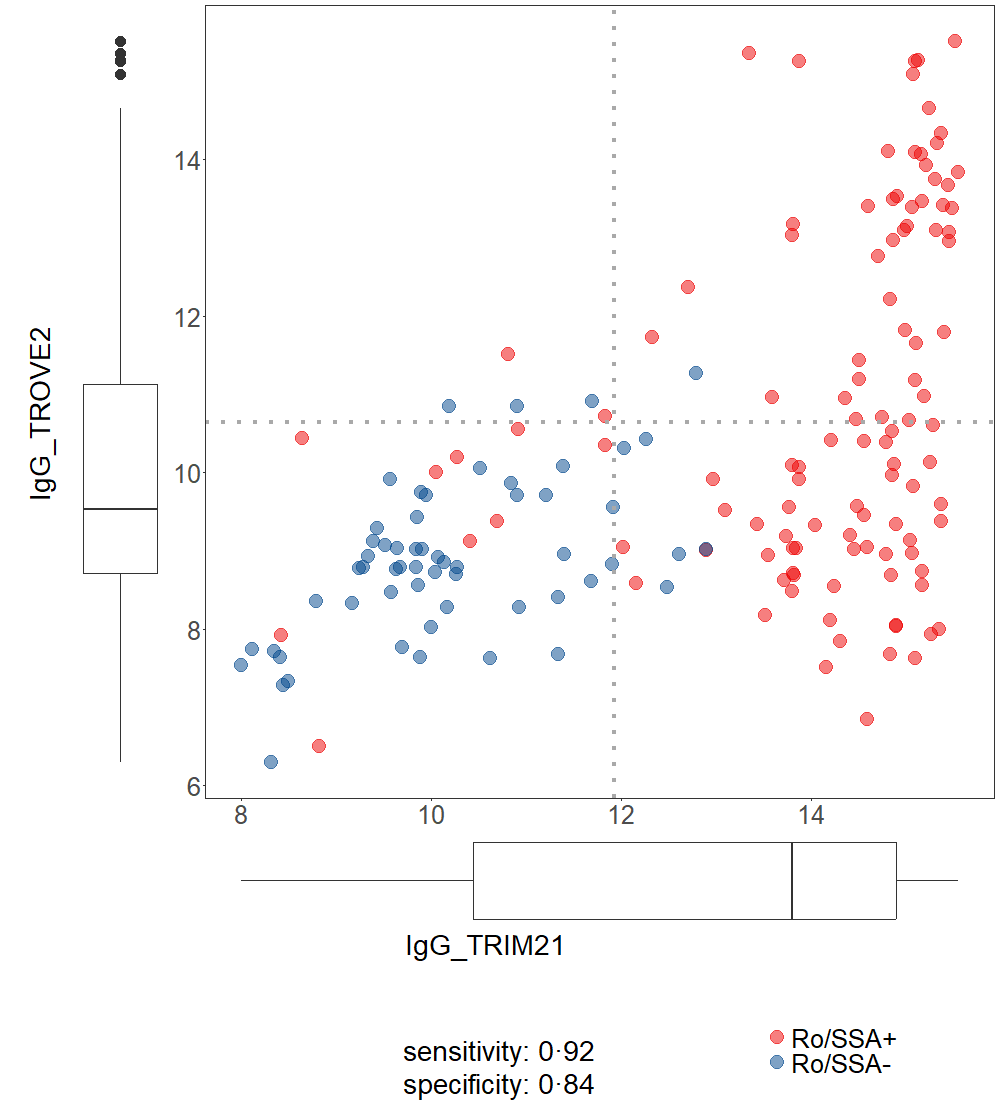
**

**Figure S1:** Scatterplot of anti-TRIM21 and anti-TROVE2 in SjD patients from Hannover (n=217) measured by Luminex antigen array. Classification of SjD patients into Ro/SSA+ and Ro/SSA- in the current study as determined by positivity for any antibody (above and/or right of the dotted lines) shows good concordance with classification by routine diagnostics (indicated by color: blue - Ro/SSA-, red - Ro/SSA+).


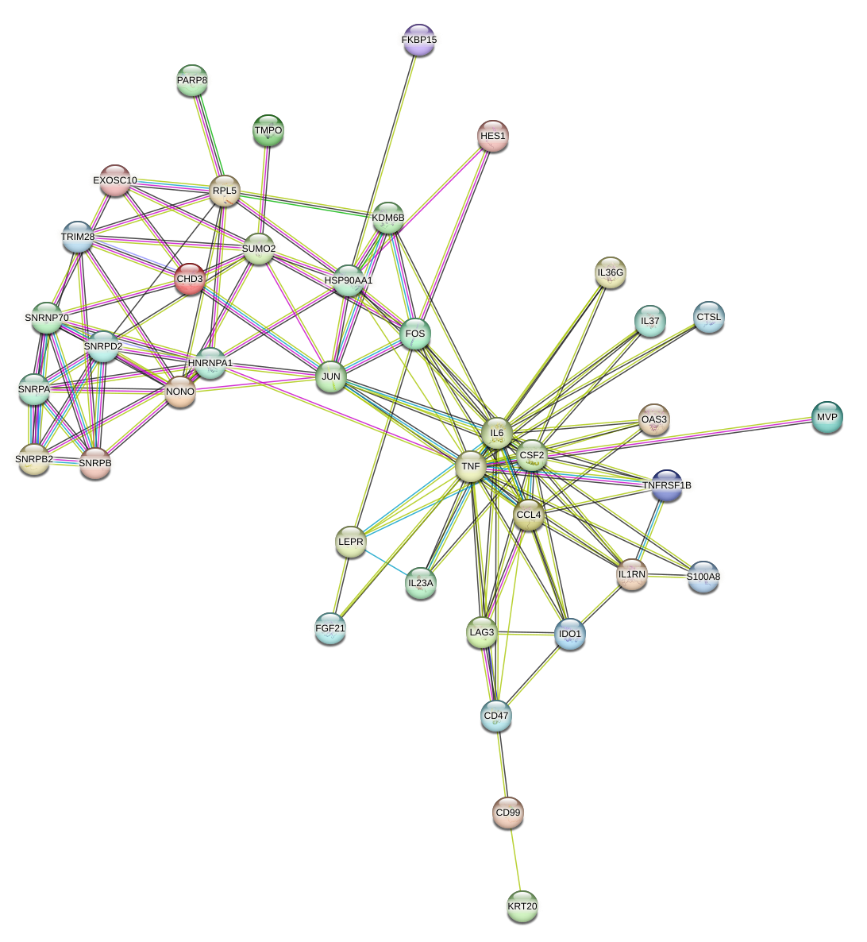


**Figure S2**: Functional protein association via STRING database. Protein interactome network were analysed, including the most common autoantibodies in primary Sjögren’s disease: CCL4, CD47, CD99, CHD3, CSF2, CTSL, EXOSC10, FGF21, FKBP15, FOS, HES1, HNRNPA1, HSP90AA1, IDO1, IL1RN, IL23A, IL36G, IL37, IL6, JUN, KDM6B, KRT20, LAG3, LEPR, MVP, NONO, OAS3, PARP8, RPL5, S100A8, SNRNP70, SNRPA, SNRPB, SNRPB2, SNRPD2, SUMO2, TMPO, TNF, TNFRFS1B and TRIM28. Edges show protein-protein associations, with interactions represented as a colored line (light blue for known interactions from curated databases, purple for known interactions determined experimentally, green for predicted gene neighborhood interactions, red for predicted gene fusion interactions, and blue for predicted gene co-occurrence interactions).

**Table S3**: Extract of functional protein interactome network via STRING database.

| **term description** | **strength** | **false discovery rate** | **matching proteins in network** |
| --- | --- | --- | --- |
| Immune system process | 0.73 | 1.32E-08 | LAG3,IL23A,OAS3,HES1,TRIM28,IL36G,IL1RN,IL37,NONO,CSF2,FOS, LEPR,HSP90AA1,CTSL,CD47,S100A8,JUN,TNFRSF1B,CD99,IL6,TNF,IDO1,CCL4 |
| Biological process involved in interspecies interaction between organisms | 0.8 | 1.18E-07 | LAG3,IL23A,OAS3,TRIM28,IL36G,IL37,NONO,CSF2,FOS,HSP90AA1,CTSL,CD47,S100A8,JUN,TNFRSF1B,IL6,TNF,IDO1,CCL4 |
| Inflammatory response | 1.08 | 1.71E-07 | IL23A,KDM6B,IL36G,IL1RN,IL37,FOS,S100A8,JUN,TNFRSF1B,IL6,TNF, IDO1,CCL4 |
| Defense response | 0.8 | 2.11E-07 | LAG3,IL23A,OAS3,TRIM28,KDM6B,IL36G,IL1RN,IL37,NONO,FOS,CD47, S100A8,JUN,TNFRSF1B,IL6,TNF,IDO1,CCL4 |
| Response to other organism | 0.8 | 8.24E-07 | LAG3,IL23A,OAS3,TRIM28,IL36G,IL37,NONO,CSF2,FOS,HSP90AA1,CD47,S100A8,TNFRSF1B,IL6,TNF,IDO1,CCL4 |
| Response to external stimulus | 0.64 | 1.18E-06 | KRT20,LAG3,IL23A,OAS3,TRIM28,IL36G,IL37,NONO,CSF2,FOS, HSP90AA1,HNRNPA1,CD47,S100A8,JUN,TNFRSF1B,IL6,TNF,IDO1,FGF21,CCL4 |
| Immune response | 0.78 | 4.16E-06 | LAG3,IL23A,OAS3,TRIM28,IL36G,IL1RN,IL37,NONO,CSF2,CTSL,CD47, S100A8,TNFRSF1B,IL6,TNF,CCL4 |
| Regulation of defense response | 0.97 | 5.53E-06 | LAG3,IL23A,OAS3,IL37,NONO,HSP90AA1,CD47,S100A8,TNFRSF1B,IL6, TNF,IDO1 |
| Positive regulation of defense response | 1.18 | 1.14E-05 | LAG3,IL23A,NONO,HSP90AA1,CD47,S100A8,IL6,TNF,IDO1 |

**
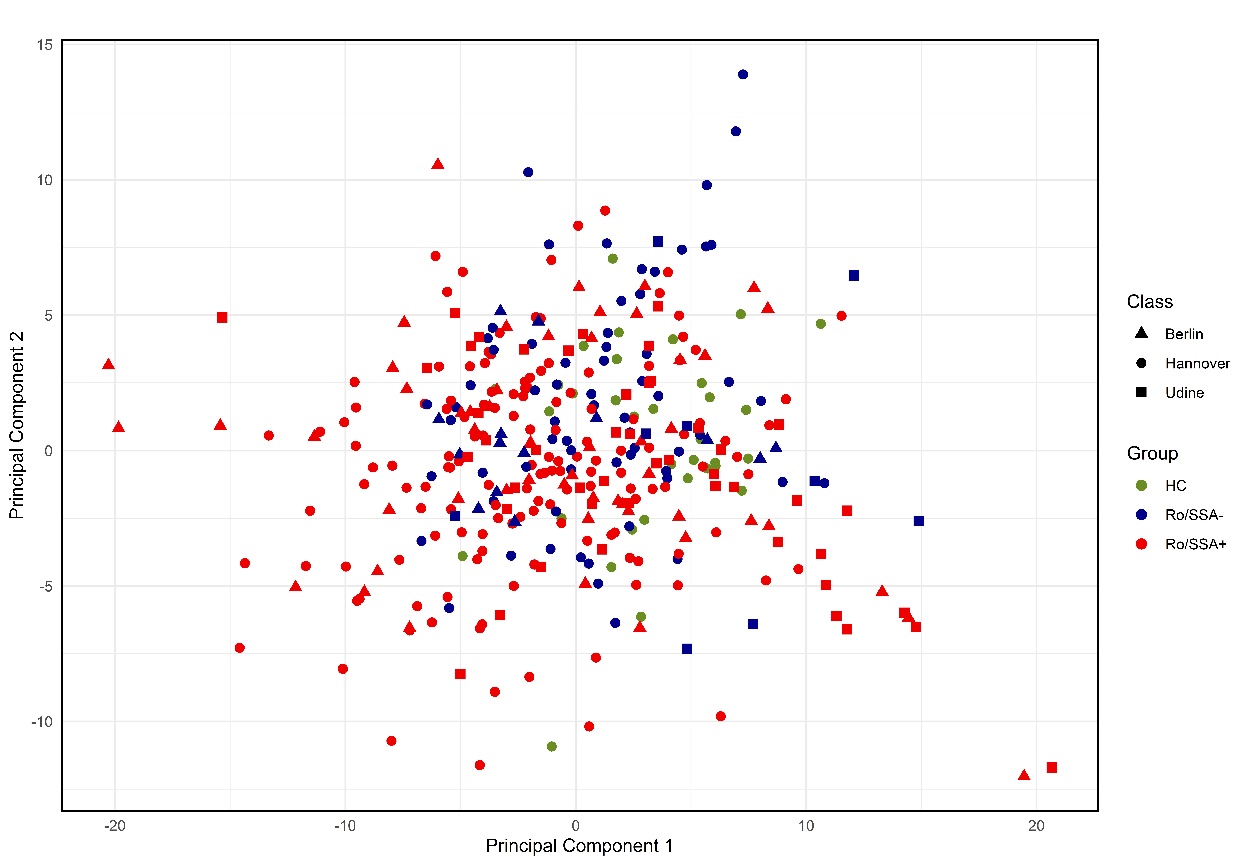
**

**Figure S3**: Autoantibody reactivity pattern in Sjögren’s disease samples illustrated by principal component analysis. Healthy individuals (HC, n=118) are given in green. Ro/SSA positive samples (n=257) are depicted in red, Ro/SSA negative samples (n=90) are given in blue. Samples from different study centers are indicated by shape.

**Table S4**: Association of autoantibodies in primary Sjögren’s disease patients (SjD, n=289) with clinical characteristics evaluated by logistic regression. P-values were adjusted by Benjamini-Hochberg procedure (q-value), whereas the coefficients refer to the adjusted odds ratio.

| **Antigen** | **Isotype** | **Parameter** | **p-value** | **q-value (adj. BH)** | **odds ratio** | **coef** | **CI.95.lower** | **CI.95.upper** |
| --- | --- | --- | --- | --- | --- | --- | --- | --- |
| RPL5 | IgG | Hypergammaglobulinemia | 0.0001 | 0.0052 | 3.0045 | 1.1001 | 0.5701 | 1.6882 |
| TRIM21 | IgG | Hypergammaglobulinemia | 0.0000 | 0.0005 | 2.0870 | 0.7357 | 0.4525 | 1.1070 |
| IL1RN | IgG | ANA | 0.0011 | 0.0374 | 2.0452 | 0.7155 | 0.3250 | 1.1882 |
| SNRPB2 | IgA | ANA | 0.0000 | 0.0019 | 1.9828 | 0.6845 | 0.3753 | 1.0300 |
| OAS3 | IgG | ANA | 0.0023 | 0.0518 | 1.9754 | 0.6808 | 0.2721 | 1.1507 |
| SSB | IgG | ANA | 0.0003 | 0.0120 | 1.8809 | 0.6318 | 0.3316 | 1.0173 |
| TRIM21 | IgA | Rheumatoid factor | 0.0000 | 0.0000 | 1.8698 | 0.6258 | 0.4796 | 0.7868 |
| AEBP1 | IgA | Chisholm Mason grade = 4 | 0.0053 | 0.0810 | 1.8358 | 0.6075 | 0.1979 | 1.0609 |
| SNRPB | IgA | Chisholm Mason grade = 4 | 0.0026 | 0.0548 | 1.8069 | 0.5916 | 0.2276 | 1.0067 |
| TRIM21 | IgA | ANA | 0.0000 | 0.0001 | 1.8046 | 0.5904 | 0.3742 | 0.8483 |
| SSB | IgG | Hypergammaglobulinemia | 0.0000 | 0.0000 | 1.8020 | 0.5889 | 0.3994 | 0.7994 |
| IL23A | IgA | ANA | 0.0102 | 0.1186 | 1.7765 | 0.5746 | 0.1516 | 1.0317 |
| TROVE2 | IgA | Rheumatoid factor | 0.0000 | 0.0000 | 1.7752 | 0.5739 | 0.4283 | 0.7339 |
| SSB | IgG | Rheumatoid factor | 0.0000 | 0.0000 | 1.7474 | 0.5582 | 0.4123 | 0.7177 |
| SPEG | IgG | Hypergammaglobulinemia | 0.0219 | 0.1932 | 1.7461 | 0.5574 | 0.0910 | 1.0503 |
| SSB | IgA | Hypergammaglobulinemia | 0.0000 | 0.0001 | 1.7348 | 0.5509 | 0.3402 | 0.7910 |
| TRIM21 | IgA | Hypergammaglobulinemia | 0.0000 | 0.0000 | 1.7322 | 0.5494 | 0.3592 | 0.7680 |
| OAS3 | IgA | ANA | 0.0014 | 0.0401 | 1.7249 | 0.5452 | 0.2287 | 0.8991 |
| IL6 | IgA | ANA | 0.0077 | 0.1015 | 1.7233 | 0.5443 | 0.1646 | 0.9693 |
| FNBP4 | IgG | ANA | 0.0167 | 0.1748 | 1.7194 | 0.5420 | 0.1246 | 1.0154 |
| CD47 | IgA | ANA | 0.0018 | 0.0471 | 1.7067 | 0.5346 | 0.2149 | 0.8904 |
| TROVE2 | IgG | Rheumatoid factor | 0.0000 | 0.0000 | 1.6963 | 0.5284 | 0.3818 | 0.6871 |
| CCL4 | IgA | Chisholm Mason grade = 4 | 0.0192 | 0.1797 | 1.6903 | 0.5249 | 0.1024 | 0.9915 |
| ZNF574 | IgA | ANA | 0.0012 | 0.0374 | 1.6901 | 0.5248 | 0.2270 | 0.8624 |
| JUN | IgG | Chisholm Mason grade = 4 | 0.0282 | 0.2068 | 1.6855 | 0.5221 | 0.0717 | 1.0161 |
| LAG3 | IgA | ANA | 0.0040 | 0.0711 | 1.6800 | 0.5188 | 0.1801 | 0.8901 |
| TROVE2 | IgA | Hypergammaglobulinemia | 0.0000 | 0.0000 | 1.6769 | 0.5169 | 0.3300 | 0.7256 |
| TROVE2 | IgG | Hypergammaglobulinemia | 0.0000 | 0.0000 | 1.6736 | 0.5150 | 0.3324 | 0.7161 |
| S100A8 | IgA | ANA | 0.0025 | 0.0538 | 1.6575 | 0.5053 | 0.1924 | 0.8499 |
| TROVE2 | IgG | ANA | 0.0002 | 0.0099 | 1.6563 | 0.5046 | 0.2605 | 0.7985 |
| IDO1 | IgA | Hypergammaglobulinemia | 0.0192 | 0.1797 | 1.6424 | 0.4962 | 0.0867 | 0.9259 |
| PMF1 | IgG | ANA | 0.0053 | 0.0810 | 1.6414 | 0.4955 | 0.1626 | 0.8625 |
| BICD2 | IgA | ANA | 0.0058 | 0.0867 | 1.6395 | 0.4944 | 0.1534 | 0.8594 |
| MVP | IgA | ANA | 0.0034 | 0.0678 | 1.6378 | 0.4933 | 0.1726 | 0.8393 |
| SNRPB2 | IgA | Hypergammaglobulinemia | 0.0012 | 0.0374 | 1.6209 | 0.4830 | 0.1999 | 0.7885 |
| IL1RN | IgA | ANA | 0.0037 | 0.0680 | 1.6189 | 0.4817 | 0.1730 | 0.8263 |
| TONSL | IgA | ANA | 0.0423 | 0.2424 | 1.5986 | 0.4691 | 0.0533 | 0.9586 |
| MVP | IgA | Hypergammaglobulinemia | 0.0084 | 0.1074 | 1.5894 | 0.4634 | 0.1293 | 0.8220 |
| FKBP15 | IgA | ANA | 0.0142 | 0.1549 | 1.5851 | 0.4607 | 0.1136 | 0.8507 |
| OAS3 | IgG | Chisholm Mason grade = 4 | 0.0360 | 0.2352 | 1.5812 | 0.4582 | 0.0420 | 0.9095 |
| SNRPD2 | IgG | Schirmer’s test | 0.0385 | 0.2374 | 0.6351 | -0.4540 | -0.8962 | -0.0283 |
| KRT20 | IgA | Pulmonary | 0.0000 | 0.0000 | 1.9371 | 0.6612 | 0.4316 | 0.9073 |
| PRR12 | IgA | Pulmonary | 0.0000 | 0.0000 | 1.9298 | 0.6574 | 0.4268 | 0.9059 |
| CTSL | IgA | Pulmonary | 0.0000 | 0.0004 | 1.7712 | 0.5717 | 0.3448 | 0.8133 |
| FAF1 | IgA | Pulmonary | 0.0000 | 0.0031 | 1.6418 | 0.4958 | 0.2679 | 0.7378 |
| SSB | IgG | Biological | 0.0000 | 0.0000 | 1.5889 | 0.4631 | 0.3153 | 0.6194 |
| TONSL | IgA | Pulmonary | 0.0000 | 0.0040 | 1.5776 | 0.4559 | 0.2418 | 0.6830 |
| SUMO2 | IgG | Biological | 0.0330 | 0.3733 | 1.5764 | 0.4552 | 0.0342 | 0.8773 |
| IL6 | IgA | Pulmonary | 0.0001 | 0.0041 | 1.5731 | 0.4531 | 0.2388 | 0.6794 |
| CTSL | IgA | Articular | 0.0001 | 0.0041 | 0.6294 | -0.4630 | -0.6962 | -0.2445 |
| SNRNP70 | IgG | Peripheral nervous system | 0.0211 | 0.3022 | 0.6257 | -0.4689 | -0.8865 | -0.0873 |
| SNRPD2 | IgG | Pulmonary | 0.0000 | 0.0011 | 0.3611 | -1.0185 | -1.4897 | -0.5936 |


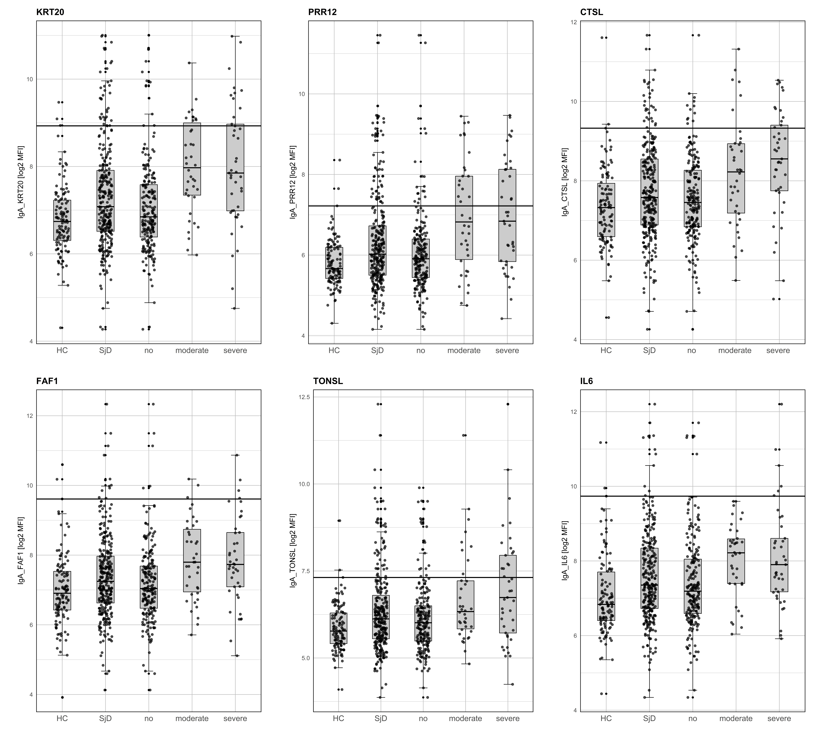


**Figure S4**: **Box and whisker plots showing the log2 MFI of pulmonary-associated markers (KRT20, PRR12, CTSL, FAF1, TONSL, IL6) in individual sample groups (HC, SjD, no lung involvement, moderate lung involvement and severe lung involvement).**
